# FT-IR Method for the Quantification of Isoflavonol Glycosides in Nutritional Supplements of Soy (*Glycine max* (L.) MERR.)

**DOI:** 10.3797/scipharm.1410-02

**Published:** 2015-03-06

**Authors:** Katharina Mulsow, Juliane Eidenschink, Matthias F. Melzig

**Affiliations:** Institute of Pharmacy, Department of Pharmaceutical Biology, Freie Universitaet Berlin, Königin-Luise-Str. 2+4, 14195 Berlin, Germany

**Keywords:** *Glycine max* (L.) MERR., Fourier Transformation Infrared, Isoflavones, Quantification

## Abstract

Due to increasing health consciousness, a lot of food supplements are sold and used. Dietary supplements of *Glycine max* (L.) MERR. are used as an alternative treatment for menopausal complaints such as hot flashes. Thereby, the effective soy compounds are the isoflavones daidzin, genistin, and glycitin. However, only the total soy extract content of the nutritional supplements is indicated. The aim of this study is to introduce a fast, efficient, and economic Fourier transformation infrared (FT-IR) spectroscopy method to quantify the active ingredients in the complex matrix of soy-based supplements. Five different nutritional supplements of *Glycine max* (L.) MERR. were purchased from a German pharmacy and were extracted with 80% aqueous methanol. A high-performance liquid chromatography (HPLC) method was used for the separation. The samples were concentrated and measured with infrared spectroscopy. An FT-IR method was established to quantify the active ingredients in the complex matrix of soy-based nutritional supplements. The partial least-squares algorithm was used to develop the method, which enabled the estimation of the content of particular isoflavones (daidzin R^2^ = 0.86, glycitin R^2^ = 0.94, genistin R^2^ = 0.96) and the quantification of the total isoflavone content (R^2^ = 0.92) despite peak overlap in the infrared (IR) spectra. The method for the quantification of the isoflavonol glycosides is precise with the standard error of prediction being 13.54%.

## Introduction

**Fig. 1:**
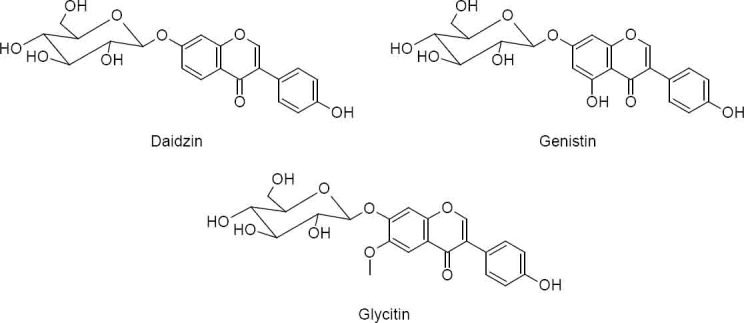
Chemical structures of the effective soy ingredients daidzin, genistin, and glycitin

Nutritional supplements of soy are used to treat menopausal symptoms. The effective ingredients are the isoflavonic glycosides daidzin, genistin, and glycitin (Fig. 1).

The isoflavones belong to the natural polyphenolic substances which have a weak affinity to both estrogen receptors [1].

*Glycine max* (L.) MERR. is an annual plant of the Fabaceae family and is used mainly for human nutrition. Health benefits such as the alleviation of menopausal symptoms, the protection against cardiovascular disease, and the protection against osteoporosis [2–4] are under discussion. Furthermore, isoflavones work as antioxidants, block angiogenesis, induce apoptosis, and reduce cell proliferation [5].

Due to the tentative character of the aforementioned health benefits, the value of supplementation with isoflavones is still under dispute.

In the German marketplace, there are various soy-based supplements, which are also available in pharmacies. They are declared as food supplements and therefore do not provide a detailed account of the extract components. Only the total content of the soy extract is indicated [6].

The quantification of isoflavonic extract compounds by HPLC and by HPTLC has been investigated by Eidenschink [7]. These methods require more time and a complex sample preparation [8].

The aim of the present work is to introduce a fast, efficient, and economic FT-IR method to analyze soy-based supplements [9]. To quantify the isoflavonic compounds, the software package Quant+ [10–12] applying the partial least-square (PLS) algorithm was used. This multivariate analysis method enables a quantitative analysis of IR spectra despite peak overlap [13]. Five soy-based supplements were examined with regard to their respective isoflavonic content by using a new FT-IR method for quantification.

## Results and Discussion

To quantify nutritional supplements easier, faster, and more cost-effective, a method was developed to determine compound concentrations from the IR spectra of plant extracts. All samples present a great quantity of data, because every value of a component is assigned to a whole spectral structure. Based on this multivariate analysis, it is possible to quantify substances in a complex matrix by near-infrared reflectance spectroscopy, for example in an herbal extract [13]. The PLS1-algorithm was used to develop this method.

By using the multivariate calibration, a correlation between the IR spectra of the samples and the corresponding concentration, which were established by HPLC, was found. Small changes in the spectra can be recognized and translated into a change of concentration with a high sensitivity. The r values for the correlation are over 0.93 (total isoflavones r = 0.96, genistin r = 0.96, daidzin r = 0.93, glycitin r = 0.97). To offset the different baselines in the spectra, the interactive baseline correction of the whole control sample was used (provided by the software program of Perkin-Elmer). Furthermore, the offset function of the Quant+ software was necessary to correct the baseline completely. The mean intensity of the whole spectrum was determined and subsequently subtracted from each intensity value of the spectrum. This procedure was chosen to achieve a baseline correction also for a small region of the spectrum. To improve the analysis, the original spectra were transformed into their first or second derivative spectra. Thereby, interaction of the baseline can be eliminated, for example drift, artifact, and constant offset. Small signals can be analyzed precisely despite background noise. Furthermore, wide signals receive a sharper increase in derivative spectra, which is necessary to develop a good method.

To obtain an applicatory method, the considered spectra were restricted to 2000–650cm^−1^, 1500–980 cm^−1^, and 1500–900 cm^−1^ for each main ingredient. In Table 1, the optimal methods for daidzin, genistin, glycitin, and for the total content of isoflavones are given.

**Tab. 1:**
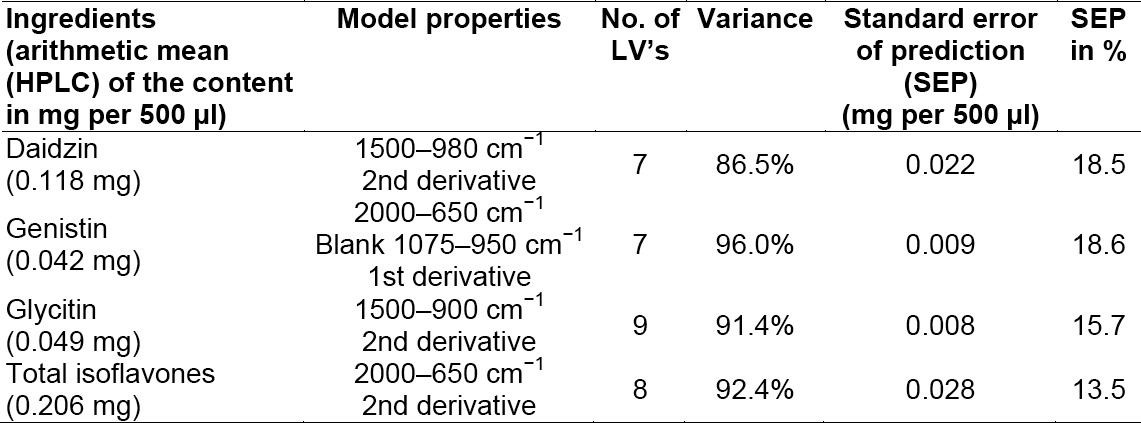
Optimal methods and corresponding properties

The omitted spectral ranges were of little informational content and therefore not included in the models.

The developed method is robust, because the quantification of all five soy-based nutritional supplements could be achieved by this method. No conspicuous structures were found in the pattern analysis of all supplements and because of that the amount of data is big and the method beneficial. The three model parameters given in Table 1 are used to assess each model.

**Fig. 2:**
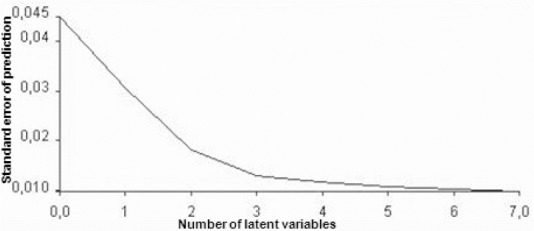
Correlation between SEP and the number of latent variables for the model of genistin

The optimal number of latent variables (no. of LVs) features a minimal standard error of prediction (SEP) and a maximum of variance. This parameter is important for the quality of the analysis. If the number of latent variables is high, the model tries to include every change in slope into the development of the model, thereby including a lot of spectral noise (overfitting). The resulting model lacks robustness. The opposite case of ‘underfitting’ provides a model, in which the correlation between the spectral data and the underlying concentrations is weak and insufficient. The correlation between SEP and the number of latent variables is shown in Fig. 2 for genistin.

The optimal number of latent variables is 7 with a minimal SEP of 0.009 mg per 500 µl. The variance should approximate the value of 100%. It is a measure of the correlation between the reference values and the reconstructed values. The correlation between the reference values and the estimated values is 92.4% for the total isoflavones.

The standard error of prediction is the extent of the reference values from the calculated values (standard deviations of the forecast). This parameter is a quantitative measure for the accuracy of the method and must be under 15% of the average of the property to allow a sufficient quantification of the unknown sample. Daidzin has a SEP of 18.51%, genistin of 18.62%, and glycitin of 15.66%, because of that the models are qualified for estimation of unknown samples [13]. The SEP is over the limit of 15%, which the Food and Drug Administration postulates [14]. The variance of 86.49% of daidzin shows great variability of the spectra. This variability of the spectra is too large in relation to the corresponding content of daidzin. The model for the total isoflavones can be used for quantification [15] and for checking the content on the nutritional herbal extracts, because the SEP is 13.54%. The SEP and the precision are sufficient for the quantification of the isoflavonol glycosides content for the nutritional supplements.

Samples are analyzed with these models to determine the content of soy isoflavones. In Table 2, the declared values of the total isoflavone content are compared with the calculated values and with the values obtained by HPLC [7].

**Tab. 2:**
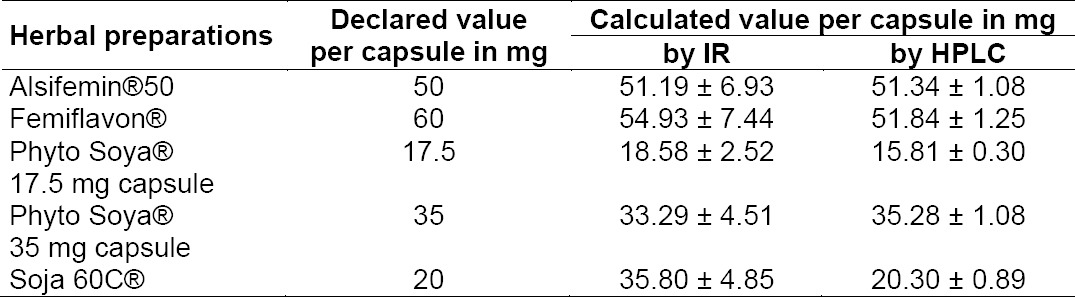
Comparison of the calculated values with the declared values of the total isoflavone content

A rough correlation of the calculated and declared values was achieved, with the exception of the Soja 60C^®^ preparation. The content of Alsifemin^®^50, Femiflavon^®^ capsules, and of Phyto Soya^®^ 35 mg capsules conform to the calculated values by HPLC. The content of Phyto Soya^®^ 17.5 mg capsules is increased with regards to the calculation by HPLC. The content (IR) of Soja 60C^®^ is 79% with regards to the declaration. The additional isoflavones of red clover present in Soja 60C^®^ could be responsible for the large deviation, because these substances can interfere with the IR spectra of soy isoflavones and the results therefore could be incorrect.

The quantitative analysis by HPLC [7] provides more evaluable results. The SEP is under 15% as shown in Table 1 and the precision is under 5% in comparison to the FT-IR method (average 13.5%). The advantage of the IR method is the easier sample preparation and the faster measurement [13].

To estimate the content of complex matrices, the FT-IR spectroscopy combined with PLS regression data analysis is an alternative to the HPLC method. The examined models can be used as fast methods to prove the content of nutritional preparations with soy and to estimate the content of the three main ingredients. The observation of other isoflavonol glycosides and aglycones are beyond the scope of this study. The study has several limitations.

To optimize the risk/benefit balance of the therapy with soy-based nutritional supplements, it is necessary to control the declaration provided by the producers.

To reach comparability between the different preparations and to select the correct daily dose, it might be advantageous to declare the content of the individual soy isoflavones.

## Experimental

### Herbal Preparations, Chemicals, and Standard Solutions

Five different nutritional supplements of *Glycine max* (L.) MERR. were purchased from a German Pharmacy: Alsifemin^®^50 (Alsitan GmbH, Germany), Femiflavon^®^ capsules (Astrid Twardy GmbH, Germany), Phyto Soya^®^ 17.5 mg capsules (Arkopharma GmbH, Germany), Phyto Soya^®^ 35 mg capsules (Arkopharma GmbH, Germany), and Soja 60C^®^ (Avitale GmbH, Germany). Methanol and acetonitrile were purchased from VWR International GmbH and were of analytical grade. Dimethyl sulfoxide was supplied by Merck KGaA.

Daidzin, genistin, and glycitin were purchased by PhytoLab (Germany). A stock solution of each standard with 80% aqueous methanol was prepared leading to a concentration of 0.244 mg mL^−1^. These stock solutions were stored in tightly sealed tubes at –15°C with the exclusion of light. For the preparation of the samples, the stock solution was added in different concentrations.

### Extraction Procedure

One hundred mg of the capsule content from Alsifemin^®^50, Phyto Soya^®^ 35 mg, and Soja 60C^®^ capsules were mixed with 1 ml dimethyl sulfoxide. Phyto Soya^®^ 17.5 mg has significantly lower declared isoflavone content; therefore 200 mg of the capsule content was used for extraction. After the addition of 24 ml 80% aqueous methanol, the solution was extracted for 20 minutes in an ultrasonic bath at 60°C.

Femiflavon^®^ capsules have a semisolid content. To obtain 100 mg of solid content, the capsule was frozen at −60°C. The extraction procedure is the same as aforementioned.

### Sample Preparation

Each extract was diluted with ultrapure water 1:1 and was used for the HPLC method.

HPLC method: The HPLC separation was performed using an Eurospher 100 C18 (250 mm x 4 mm, 5 µm) with a WellChrom HPLC-Pump K-1001, a WellChrom Solvent Organizer K-1500, a WellChrom LAMP K-2701, and a WellChrom DAD K-2700. EuroChrom 2000 Basic Edition was used as software. The mobile phase A consisted of 50% aqueous acetonitrile and the mobile phase B of ultrapure water.

For the separation of the Alsifemin^®^50, Phyto Soya^®^ 35 mg, Phyto Soya^®^ 17.5 mg, and Femiflavon^®^ extracts, a gradient elution was applied (acetonitrile 50% / water gradient from 20/80 to 50/50 over 25 min). After 30 min, the acetonitrile 50% / water ratio was set to 90/10, and after 32 min set to 20/80. Because the Soja 60C^®^ extract contains red clover and soy isoflavones, the gradient elution of the HPLC method was modified to reach separation of the compounds (acetonitrile 50% / water gradient from 20/80 to 50/50 over 15 min, after 25 min the acetonitrile 50% / water ratio was set to 90/10, and after 32 min set to 20/80).

The flow rate was 1.2 ml/min. UV detection was operated at 260 nm.

### Quantification by FT-IR

To receive a calibration range, the untreated extracts and extracts with added isoflavones daidzin, genistin, and glycitin were measured (Table 3).

**Tab. 3:**
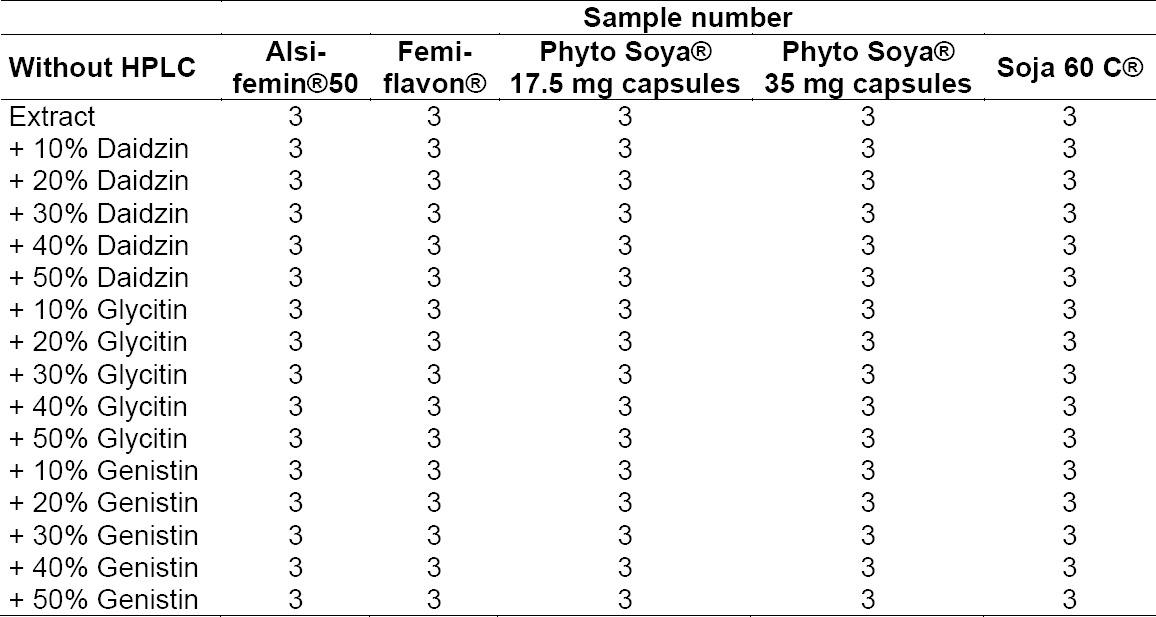
Extract samples with and without added isoflavon standard solutions

The solvent was removed with the SpeedVac Concentrator Savant SPD111V with a high vacuum at 60°C. Furthermore, extracts with only two isoflavons were used for the calibration range (Table 4).

**Tab. 4:**
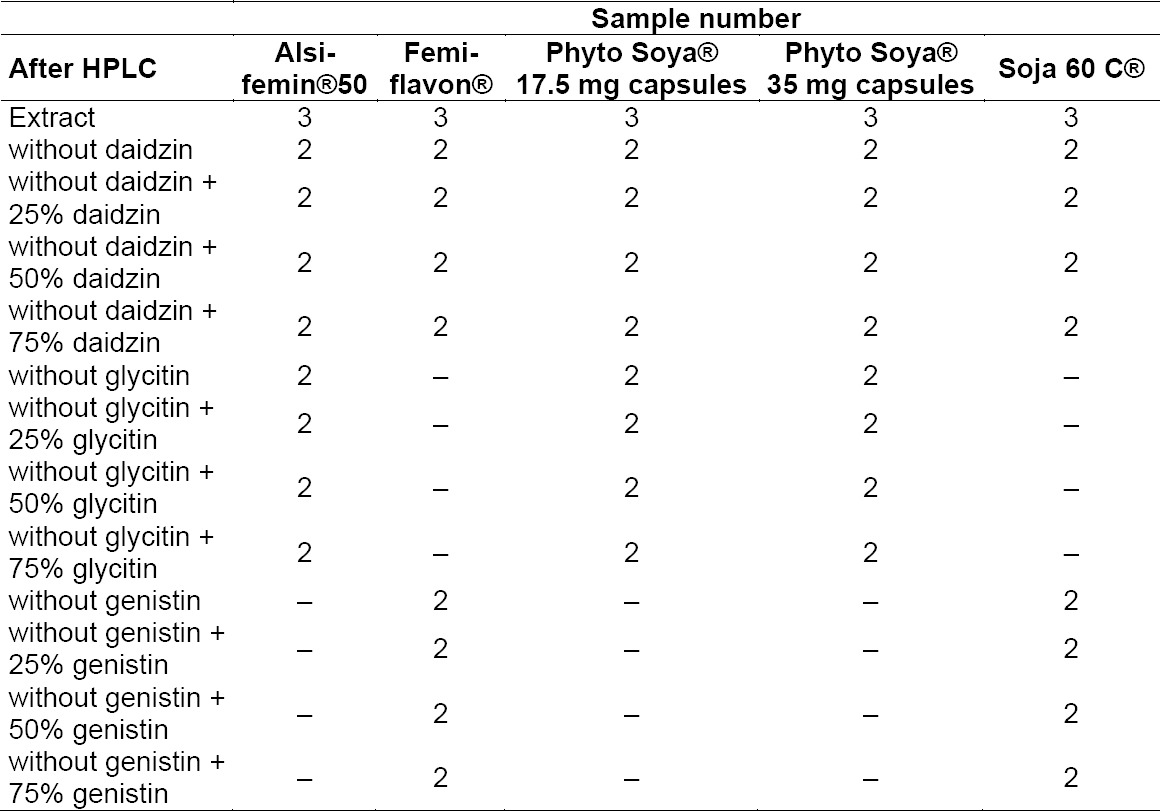
Samples after separation by the HPLC method

The ingredients were excluding by the mentioned HPLC-method. An amount of 500 µl of the sample was needed for the measurement. Therefore, the HPLC separation had to be repeated five times to receive one sample with the volume of 500 µl. These obtained samples were used untreated and were spiked with one of the three isoflavon standard solutions. To remove the solvent, the SpeedVac Concentrator Savant SPD111V with a high vacuum at 60°C was used.

For the analysis by FT-IR spectroscopy, 335 samples were prepared to develop an optimized model (Tables 3 and 4).

The dried extracts were concentrated and measured with infrared spectroscopy (Perkin Elmer precisely Spectrum 100 with a Universal Diamond/ZnSE-ATR sampling accessory). Data analysis was performed using the Quant+ software. The IR spectra were scanned and a PLS-regression was implemented. For the multivariate calibration, special regions of the spectra were selected, because the structure of the isoflavonic glycosides influenced only particular sections. Furthermore, a baseline correction, the formation of a first or second derivative by using the Savitzky-Golay function over a 9-measuring point interval, and the standard normal variate (SNV) can be chosen. The SNV was used to avoid multiplicative interferences by light dispersion and particle size [10].
